# Effect of repeat human blood feeding on *Wolbachia* density and dengue virus infection in *Aedes aegypti*

**DOI:** 10.1186/s13071-015-0853-y

**Published:** 2015-04-24

**Authors:** Hilaria E Amuzu, Cameron P Simmons, Elizabeth A McGraw

**Affiliations:** School of Biological Sciences, Monash University, Clayton, Melbourne, Victoria Australia; Department of Microbiology and Immunology, University of Melbourne, Parkville, Melbourne, Victoria Australia

**Keywords:** *Wolbachia*, Dengue, *Aedes aegypti*, Blood feeding

## Abstract

**Background:**

The introduction of the endosymbiotic bacterium, *Wolbachia* into *Aedes aegypti* populations is a novel approach to reduce disease transmission. The presence of *Wolbachia* limits the ability of the mosquito to transmit dengue virus (DENV) and the strength of this effect appears to correlate with *Wolbachia* densities in the mosquito. There is also some evidence that *Wolbachia* densities may increase following the consumption of a bloodmeal. Here we have examined whether multiple blood feeds lead to increases in density or associated changes in *Wolbachia*-mediated blocking of DENV.

**Methods:**

The *Wolbachia* infected *Aedes aegypti* mosquito line was used for the study. There were three treatment groups; a non-blood fed control, a second group fed once and a third group fed twice on human blood. All groups were orally infected with DENV-2 and then their midguts and salivary glands were dissected 10–11 days post infection. RNA/DNA was simultaneously extracted from each tissue and subsequently used for DENV RNA copies and *Wolbachia* density quantification, respectively.

**Results:**

We found variation between replicate vector competence experiments and no clear evidence that *Wolbachia* numbers increased in either the salivary glands or remainder of the body with feeding and hence saw no corresponding improvements in DENV blocking.

**Conclusions:**

*Aedes aegypti* are “sip” feeders returning often to obtain bloodmeals and hence it is important to assess whether repeat blood feeding improved the efficacy of *Wolbachia*-based DENV blocking. Our work suggests in the laboratory context when *Wolbachia* densities are high that repeat feeding does not improve blocking and hence this ability should likely be stable with respect to feeding cycle in the field.

## Background

Dengue is a re-emerging infectious disease caused by dengue viruses (DENV) and is transmitted by mosquitoes of the genus *Aedes* including *Ae. aegypti* and *Ae. albopictus,* with the former being the principal vector. It is endemic in over 100 countries in Asia, The Pacific, Africa, The Americas and The Caribbean with 390 million infections annually [1]. The disease is severely debilitating with symptoms ranging from mild flu with rash (dengue fever) to a severe and sometimes fatal disease (dengue hemorrhagic fever) [2]. There is no licensed vaccine and no specific treatment for dengue fever. The difficulty in developing a vaccine has been mainly attributed to the existence of the four different serotypes (DENV 1–4) and the fact that the characteristics of protective immunity are not well understood [3].

In response, there is a growing focus on novel control approaches including the maternally transmitted endosymbiotic bacterium *Wolbachia pipientis* (Class: Alphaproteobacteria; Order: Rickettsiales). It is naturally found in over 40% of insects [4]. However *Ae. aegypti* does not naturally harbour these bacteria unlike 28% of other mosquito species including *Culex quinquefaciatus, Culex pipiens* and *Ae. albopictus* [5]. In the last decade three different strains of *Wolbachia* have been successfully introduced into *Ae. aegypti* where they form stably inherited infections. These are *w*Mel and *w*MelPop-CLA from *Drosophila melanogaster* and *w*AlbB from *Ae. albopictus* [6-8]. These transinfections were carried out with the hope of finding a means to use *Wolbachia* for vector control. The symbiont gained initial attention for this purpose as *Wolbachia* induces a phenomenon called cytoplasmic incompatibility which results in inviable eggs when infected males mate with uninfected females or a female infected with a different strain [9]. As *Wolbachia* is maternally transmitted it is able to quickly invade or replace wild populations, an attractive characteristic for biocontrol [8,10].

An unexpected discovery was made after the creation of the transinfected lines of *Ae. aegypti*. The presence of *Wolbachia* was shown to interrupt/block replication and hence transmission of various pathogens transmitted by mosquitoes including DENV [8,11]. The mechanism of pathogen blocking is poorly understood but some studies have demonstrated the involvement of competition for nutrient(s) between the virus and the bacteria such as cholesterol [12]. Other studies reveal that the presence of *Wolbachia* up-regulates the immune effectors of the host thereby enabling it to resist subsequent viral infection; that is ‘immune priming’ [13,14].

In 2011 open field releases of *w*Mel infected *Ae. aegypti* were carried out in the Cairns communities of Yorkeys Knob and Gordonvale in Australia to assess the dispersal of *Wolbachia. Wolbachia* infection frequencies in these areas reached fixation after 12 weeks of release where they have subsequently remained [15]. Biocontrol of dengue through *Wolbachia* is proving to be sustainable, less expensive and more specific in approach than other vector control strategies [16]. Since 2013, ongoing releases are being carried out in Vietnam and Indonesia where the ability of *Wolbachia* to reduce dengue virus transmission in the human population can actually be tested given the endemicity of the disease in these countries.

*Wolbachia* is found at different densities in various tissues of the mosquito body [17]. Studies by Bian and colleagues [18] have shown that the inhibition/blocking of DENV by *Wolbachia* varied in different tissues. At the cellular level, it has been observed that the higher the *Wolbachi*a density per cell, the greater the degree of viral inhibition [19]. *Ae. albopictus* which is naturally infected with *w*AlbB and *w*AlbA strain of *Wolbachia* has lower density of the bacteria in somatic tissues compared to *Ae. aegypti* transinfected with *w*AlbB and hence it does not normally block dengue [20]. It is therefore thought that the strength of blocking may be explained by either the tissue distribution or density of *Wolbachia* [11,20].

*Wolbachia* density in moth and beetle is known to be influenced by several factors including host genotype [21,22] and environmental conditions such as temperature in wasps [23]. It has also been demonstrated in *Ae. albopictus* larvae that nutritional restrictions lead to low *Wolbachia* density [24]. Furthermore, preliminary data suggests that *Wolbachia* densities increase inside whole mosquitoes when fed sheep’s blood in the laboratory [25]. This effect therefore has the potential to improve dengue blocking over the lifetime of the mosquito and further reduce transmission to humans. Therefore, we sought to investigate the relationship between human blood feeding, *Wolbachia* densities and DENV blocking in the midgut and salivary glands, the tissues necessary for infection and transmission of DENV in the mosquito, respectively [26]. In this study, we used *Ae. aegypti* mosquitoes infected with *w*Mel *Wolbachia* [8] sampled from field release sites [15] which has been denoted *w*Mel.F mosquitoes [25] and found that repeat human blood feeding did not significantly increase *Wolbachia* density in the midgut and salivary glands nor did it alter blocking ability against DENV.

## Methods

### Ethics statement

Ethical approval was obtained from the Monash University Human Research Ethics Committee (permit CF11/0766-2011000387). All volunteers gave written informed consent prior to taking part in this study.

### Rearing of mosquitoes

Two *Ae. aegypti* mosquito lines were used for the experiments. These were the outcrossed *Ae. aegypti* mosquitoes transinfected with the *w*Mel *Wolbachia* strain [8] sampled from the field release sites in Cairns, Australia [15] and *Ae. aegypti* not infected with *Wolbachia* from neighbouring communities. These two mosquito lines were denoted *w*Mel.F and Wildtype respectively [25]. The mosquitoes were reared under standard conditions of 25°C temperature, 65% relative humidity and photoperiod 12 hours light: dark. The larvae were fed TetraMin® (Melle, Germany) fish food *ad libitum* while the adults were kept on 10% sucrose.

### Human blood feeding of mosquitoes

The *w*Mel.F and Wildtype *Ae.aegypti* mosquitoes were concurrently reared for this experiment. There were three treatment groups for each of the two lines: a control group that was not fed on human blood (Unfed), a second group fed only once on human blood (Fed 1×) and a third group fed twice on human blood (Fed 2×). Apart from the mosquitoes in the Unfed group, all adult female mosquitoes in the Fed 1× and Fed 2× groups were first fed directly on human blood 5 days after eclosion. The mosquitoes which did not feed were sorted the next day and discarded. The rest of the mosquitoes in the third group (Fed 2×) were fed a second time 7 days after the first feed (that is 12 days post eclosion). Oviposition cups were provided after each bloodmeal for egg laying. One single human served as a bloodmeal source for both lines used in the experiment.

### Dengue oral feeds and dissections of salivary glands and midguts

Frozen DENV-2, ET300 (collected from a patient in East Timor in 2000) with titre 10^6^ PFU/ML was propagated as per a previously reported method [19]. Virus passage number 6 was used for all experiments. The virus was mixed with defibrinated sheep blood in the ratio 1:1 and then fed simultaneously to all three treatment groups of both *w*Mel.F and Wildtype mosquitoes 19 days post eclosion using a pig’s intestine as a membrane. All mosquitoes were starved 24 hrs prior to feeding. The mosquitoes were fed for three hours and those which did not feed were discarded the next day. Ten to 11 days post infection (29–30 days post eclosion) the salivary glands and midguts of each mosquito in all the three treatment groups in the two mosquito lines were dissected in 1X PBS after anaesthetising them on ice. Each tissue was kept separately in 200 ul of TRIzol® (Life Technologies, Carlsbad, California, USA) and then stored at −80°C for simultaneous RNA/DNA extractions for DENV RNA copies/*Wolbachia* density quantification. A total of 20–24 mosquitoes were dissected from each of the three treatment groups of each line. To ensure that the results obtained were reproducible, the entire experiment was then replicated/repeated and denoted replicates A and B.

As mentioned previously, all three treatment groups of the two mosquito lines were maintained under the same standard conditions and fed 10% sucrose throughout the experiment. Hence any resulting changes that may occur in *Wolbachia*/DENV RNA copies will be the effect of bloodmeal alone.

### RNA/DNA extractions

RNA/DNA was simultaneously extracted from each salivary gland, midgut and the remainder of the mosquito body using the TRIzol® method from Invitrogen (Life technologies, Carlsbad, California, USA). Extracted total RNA was stored at −80°C and the DNA at −20°C prior to cDNA synthesis for DENV and *Wolbachia* quantification respectively.

### qRT-PCR quantification of DENV

Viral cDNA synthesis was carried out on the RNA using the method of Moreira *et al.* [11] followed by DENV quantification using HEX labelled probe and primers designed for the 3’UTR region by Warrillow *et al.* [27]. DENV RNA copy numbers were calculated using a standard curve for DENV-2 and was constructed as in Moreira *et al.* [11]. All qPCR reactions were carried out in LightCycler480 (Roche,Applied Science, Switzerland). The cycling conditions were 95°C for 5min, followed by 45 amplification cycles of 95°C for 10s, 60°C for 15s, 72°C for 1s and a final cooling step of 40°C for 10s. Each tissue was run in duplicates and a sample was called uninfected (copy number =0) when both technical replicates come out negative.

### *Wolbachia* density quantification

The *Wolbachia* surface protein, *wsp* was quantified in reference to the housekeeping gene *Rps*17 of the mosquito [28,29]. Taqman multiplex qPCR was carried out in Lightcycler480 (Roche, Applied Science, Switzerland) following the protocol of Frentiu *et al.* [25]. There were 2 technical replicates for each dissected tissue. The *wsp/Rps*17 ratio was calculated using the advanced relative quantification algorithm software in LightCycler480 (Roche Applied Science, Switzerland).

### Statistical analysis

The number of DENV infected and uninfected tissues were compared between treatment groups in each of the two replicate experiments (A and B) using Fisher’s exact test. DENV RNA copy numbers between treatment groups in each of the two replicate experiments (A and B) were compared using Mann Whitney test. Treatments were only compared within mosquito lines. Differences in *Wolbachia* density between treatment groups in each of the two replicate experiments (A and B) were compared using Mann Whitney test. All statistical tests were carried out in Graphpad prism Version 6.04 (San Diego, California, USA).

## Results and discussion

The mechanisms involved in *Wolbachia*-mediated DENV blocking are not well understood but to date appear to be comprised of an interplay of a host of factors including competition for limited nutritional resources and host immunity [12-14]. *Wolbachia* density also appears to be positively correlated with the level of pathogen blocking [30]. In both mosquito cell lines [19,20] and in whole mosquitoes [8] higher *Wolbachia* infections show increased DENV blocking. *Wolbachia* density is likely regulated by a number of factors including host genetic background, environmental conditions and nutrient availability [21,22,24]. Increased *Wolbachia* density was observed following a single bloodmeal in *w*Mel infected mosquitoes collected from the field post-release [25]. If a relationship exists between *Wolbachia* densities and blood feeding, then multiple blood feedings on humans in the field could lead to greater viral inhibition over the life of the mosquito. This is particularly relevant in the case of *Ae. aegypti* that return to feed frequently on human hosts [31-33]. Here we show that multiple blood feeding events do not increase the *Wolbachia* densities in a predictable manner nor affect DENV RNA copies in key tissues (midguts and salivary glands) that serve as checkpoints or barriers to infection and transmission [26].

### DENV infection rates

In the two replicate experiments (A and B), fewer *w*Mel.F mosquito tissues were infected with DENV as compared to the Wildtype mosquitoes (Tables 1 and 2) as predicted [8,11]. The level of blocking by *w*Mel.F mosquitoes was shown to be improved compared to the level present in the original laboratory colony (MGYP2 mosquitoes) prior to release [25], showing support for the long-term sustainability of *Wolbachia* mediated biocontrol against DENV in the field. Infection rates, however, did not change in a predictable fashion following repeated human blood feeding for any of the tissues studied in both replicate A and B (Tables 1 and 2).

**Table 1:** **DENV-2 infection rates (%) for replicate A tissues of** ***w*** **Mel.F and Wildtype mosquitoes**

**Blood feeding status**	**Salivary glands infections (N)**		**Midguts infections (N)**		**Body infections (N)**	
	***w*** **Mel.F**	**Wildtype**	***w*** **Mel.F**	**Wildtype**	***w*** **Mel.F**	**Wildtype**
Unfed	23 (22)	83 (24)	32 (22)	96 (24)	22 (22)	83 (24)
Fed 1x	8 (24)	85 (20)	13 (24)	95 (20)	8 (24)	85 (20)
Fed 2x	0 (23)*	54 (24)	30 (23)	58 (24)**	0 (23)*	54 (24)

Fisher’s exact tests: *Fed 2x v Unfed, P < 0.05; **Fed 2x v Unfed and Fed 2x v Fed 1x, P < 0.05. Comparisons were done between treatment groups within mosquito lines.

**Table 2:** **DENV-2 infection rates (%) for replicate B tissues of** ***w*** **Mel.F and Wildtype mosquitoes**

**Blood feeding status**	**Salivary glands infections (N)**		**Midguts infections (N)**		**Body infections (N)**	
	***w*** **Mel.F**	**Wildtype**	***w*** **Mel.F**	**Wildtype**	***w*** **Mel.F**	**Wildtype**
Unfed	0 (24)	63 (24)	21 (24)	83 (24)	4 (24)	63 (24)
Fed 1x	0 (24)	46 (24)	8 (24)	71 (24)	4 (24)	46 (24)
Fed 2x	0 (23)	83 (24)*	4 (23)	92 (24)	0 (23)	83 (24)*

Fisher’s exact tests: *Fed 2x v Fed 1x, P < 0.05. Comparisons were done between treatment groups within mosquito lines.

### *Wolbachia* density in dissected tissues

**Figure 1:**
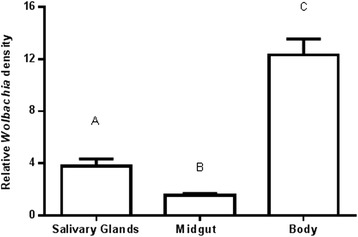
*Wolbachia* density in salivary gland, midgut and body of *w*Mel.F mosquitoes. Significantly more *Wolbachia* was found in the remainder of the mosquito body compared to the salivary glands and midguts (P < 0.0001). Salivary glands had significantly more *Wolbachia* than the midguts (P = 0.0009). Y-axis shows ratio of *wsp*/*Rps*17. Letters denote distinct statistical groups and error bars are standard error of the mean of 16–17 biological replicates.

The *Wolbachia* densities were significantly different in the tissues examined. The midgut had the lowest *Wolbachia* density and was 2.43-2.5 fold lower than that of salivary glands. The mosquito body had the highest density and was 3.3-5.3 fold higher than that of salivary glands and 7.9-13 fold higher than that of the midgut (Figure 1). The body included the ovaries and therefore was expected to have higher densities of *Wolbachia*. These findings are consistent with previously published characterisations of the *w*AlbB and *w*AlbA strains present in *Ae. albopictus* and the *w*AlbB strain stably transinfected into *Ae. aegypti* [20].

### Relationship between DENV RNA copies and *Wolbachia* density - salivary glands

**Figure 2:**
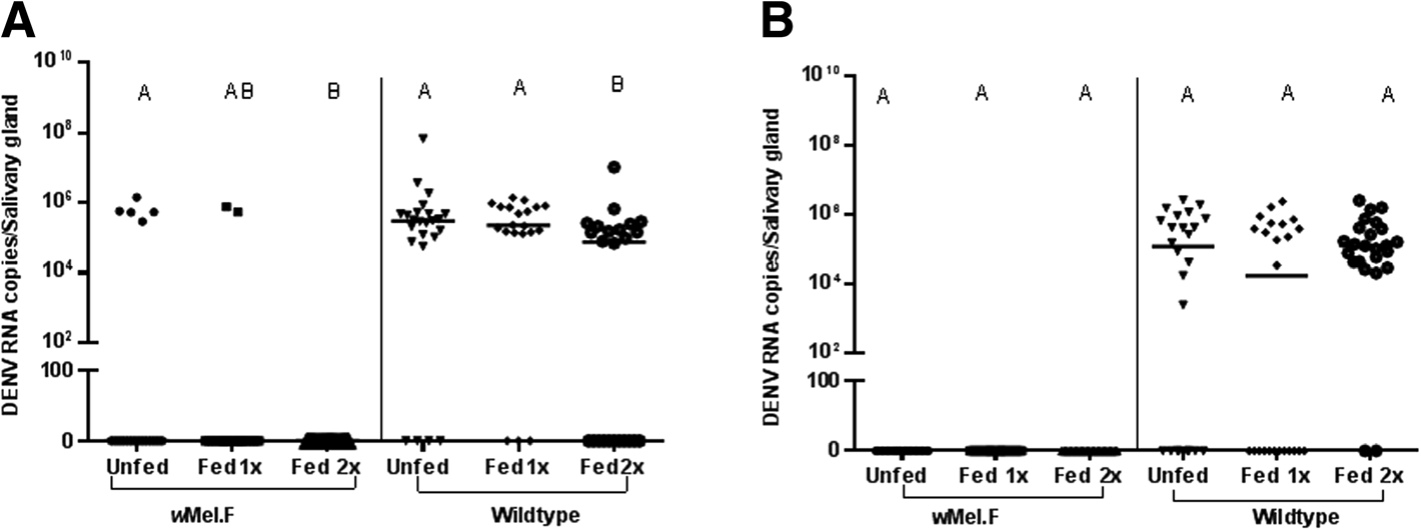
DENV-2 RNA copies in salivary glands of *w*Mel.F and Wildtype mosquitoes. In replicate **A**, *w*Mel.F mosquitoes fed twice on human bloodmeal (Fed 2x) prior to being infected had complete viral blocking 10–11 dpi. This was significantly lower (P = 0.02) than the *w*Mel.F mosquitoes which were not fed on human bloodmeal (Unfed) prior to being infected. The Wildtype mosquitoes which had two repeat human bloodmeals (Fed 2x) had significantly lower copies of DENV-2 RNA compared to those which did not have human bloodmeal (Unfed) (P = 0.005) and those which had only one human bloodmeal (Fed 1x) (P = 0.005). In replicate **B**, there was complete DENV-2 blocking in the *w*Mel.F salivary glands in all three treatment groups and in the Wildtype mosquitoes, the effect of repeat blood feeding was not significant. Comparisons were made within mosquito lines and across treatment groups. Letters represent distinct statistical groups. Bars denote medians and each point represents individual salivary gland.

**Figure 3:**
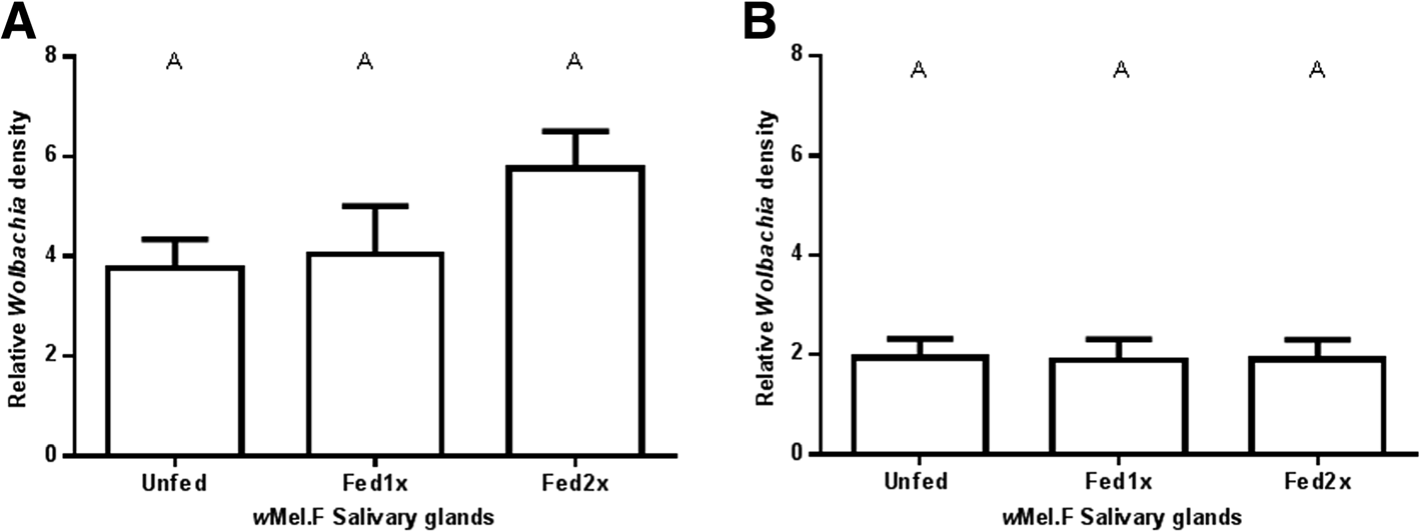
*Wolbachia* density in *w*Mel.F mosquito salivary glands. *w*Mel.F mosquitoes fed once and twice on human bloodmeals (Fed 1x and Fed 2x) prior to being challenged with DENV-2 did not have a significant change in *Wolbachia* density in both replicates **A** and **B** compared to the Unfed controls which were not blood fed. Y-axis shows ratio of *wsp*/*Rps*17. Letters represent distinct statistical group. Error bars are standard error of the mean of 11–19 salivary glands.

There was little (Figure 2A) to no (Figure 2B) DENV infection of the salivary glands in the presence of *w*Mel making it difficult to assess the effects of repeat feeding for this tissue. In replicate A, however, where DENV was present, *w*Mel.F mosquitoes that fed twice exhibited greater inhibition than the controls (Figure 2A). Regardless, *Wolbachia* densities did not increase with repeat blood feeding in either replicate experiment (Figure 3A, B). Hence the complete inhibition of DENV after the second feed in replicate A may not be explained by *Wolbachia* density. It is possible that immunity of the mosquito may have been improved by availability of nutrients through blood feeding [34,35] but this is not consistent with the lack of an effect in the Wildtype (Figure 2B). Alternatively, the positive result in replicate A may be due to a synergy between *Wolbachia* and blood feeding or be an artefact of small sample sizes.

### Relationship between DENV RNA copies and *Wolbachia* density - midgut

**Figure 4:**
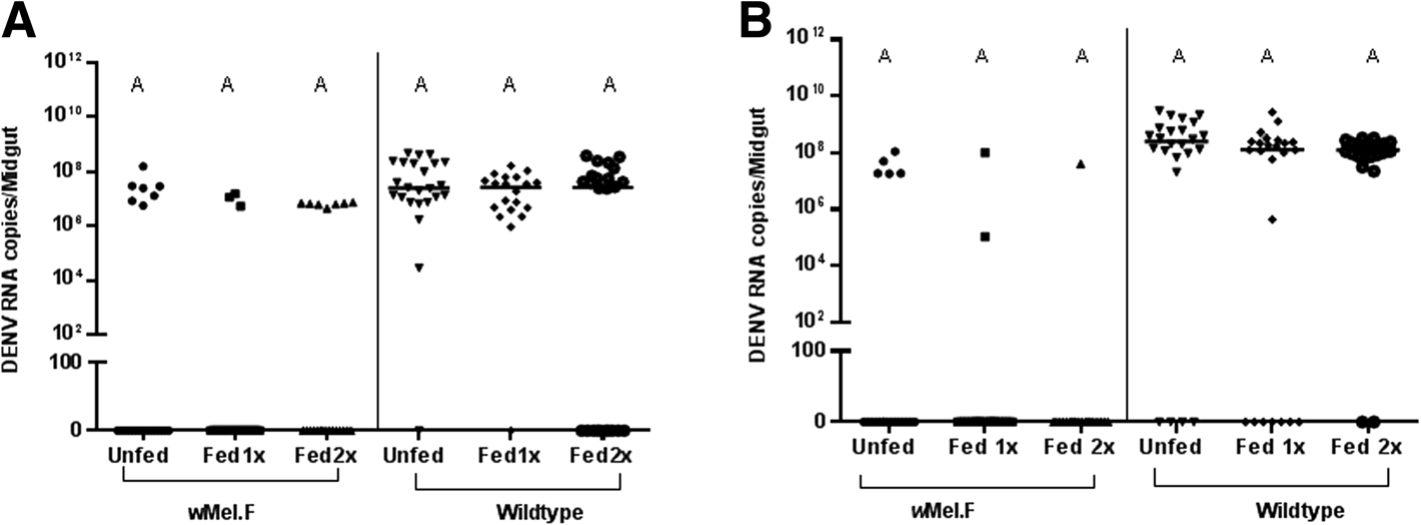
DENV-2 RNA copies in midgut of *w*Mel.F and Wildtype mosquitoes. DENV-2 RNA copies in midguts of *w*Mel.F and Wildtype mosquitoes fed once and twice on human bloodmeal (Fed 1x and Fed 2x) did not change significantly compared to those which were not blood fed (Unfed) in both replicates **A** and **B**. Comparisons were made within mosquito lines and within treatment groups. Letters represent distinct statistical group. Bars denote medians and each point represents an individual midgut.

**Figure 5:**
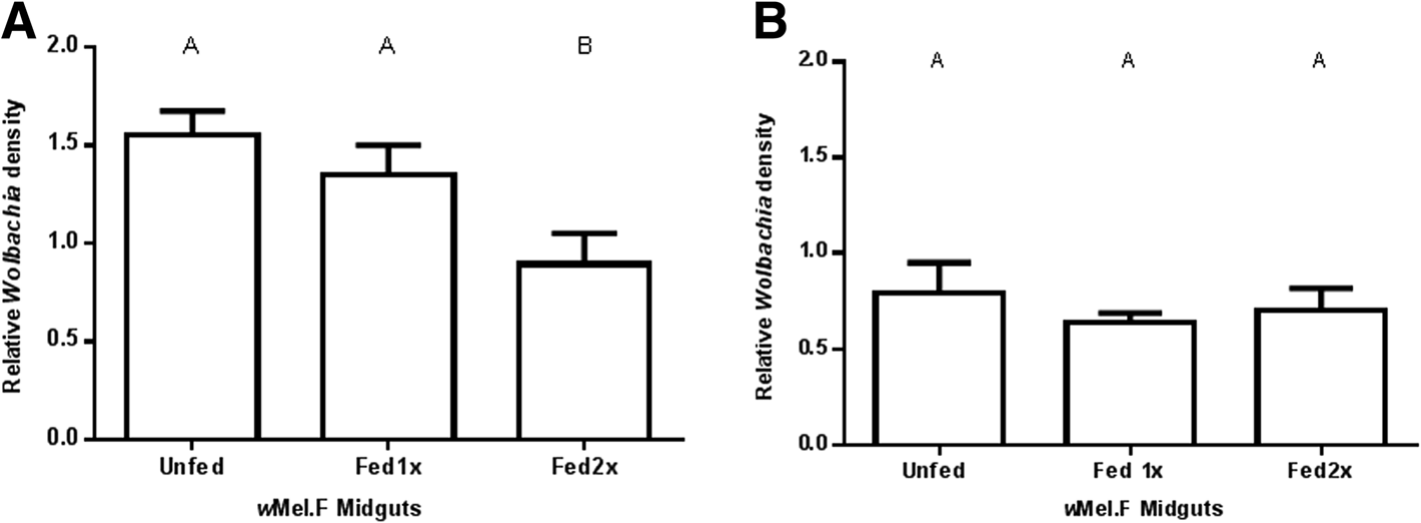
*Wolbachia* density in *w*Mel.F mosquito midguts. In replicate **A**, midgut *Wolbachia* density in *w*Mel.F mosquitoes decreased in mosquitoes fed twice on human bloodmeal (Fed 2x) before being challenged with DENV-2 compared to those which were not blood fed (Unfed) (P = 0.002) and those fed only once on human blood (Fed 1x) (P = 0.03). However in replicate **B**, there was no significant change in *Wolbachia* density across treatment groups. Y-axis shows ratio of *wsp*/*Rps*17. Letters represent distinct statistical groups. Error bars are standard error of the mean of 14-21individual midguts.

Repeat blood feeding did not significantly affect DENV RNA copies in the midgut of *w*Mel.F infected mosquitoes in replicate A (Figure 4A) and in replicate B (Figure 4B), DENV infection rates were lower, making comparisons of viral RNA concentration difficult to assess. There were no consistent effects of blood feeding on *w*Mel density in the midgut, but there was an unexpected significant decrease in *Wolbachia* density after the second bloodmeal compared with controls and those fed one time for replicate A (Figure 5A) but not replicate B (Figure 5B). This effect was also observed in *w*Flu from *Ae. fluviatilis* where the midgut *Wolbachia* density of blood fed individuals were consistently lower compared to sugar-fed females [36]. Surprisingly, this change did not have an effect on DENV infection or RNA copies in the *w*Mel.F midgut. It should be determined if there is a threshold *Wolbachia* density in mosquitoes below which viral blocking is interrupted or if densities in only a limited set of tissues are predictive of blocking ability. Repeat feeding had no consistent effect on DENV RNA copies in the midgut of Wildtype mosquitoes (Figure 4).

### Relationship between DENV RNA copies and *Wolbachia* density - body

**Figure 6:**
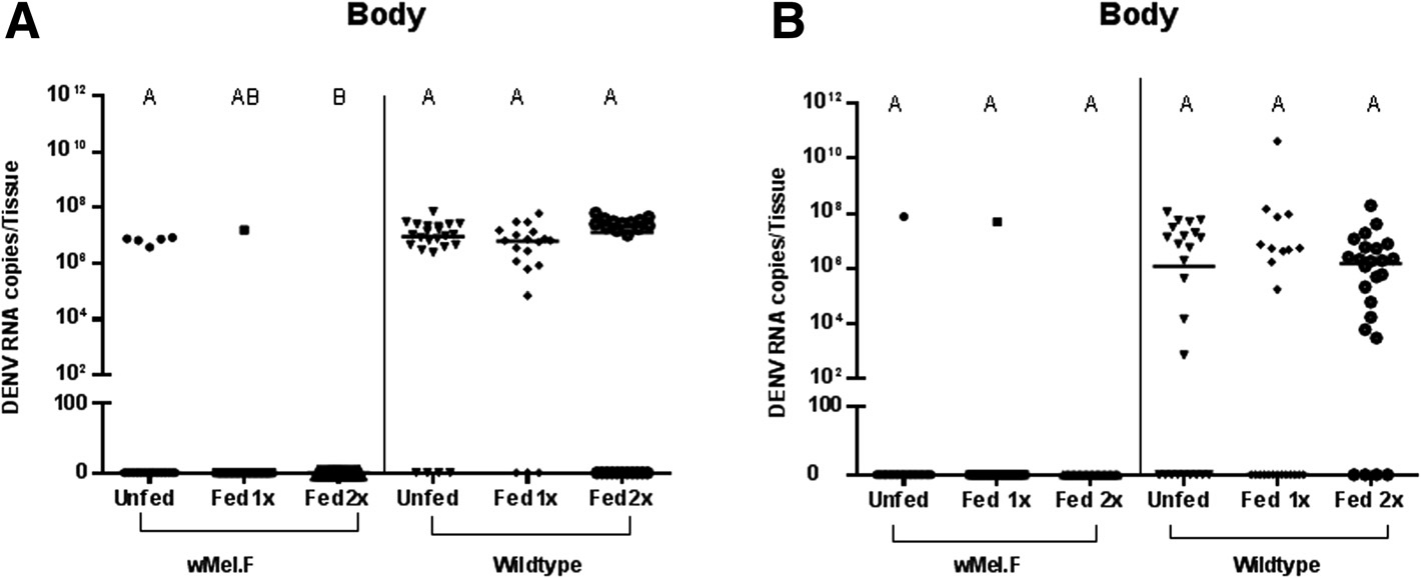
DENV-2 RNA copies in the body of *w*Mel.F and Wildtype mosquitoes. In replicate **A**, *w*Mel.F mosquitoes that fed twice on human bloodmeal (Fed 2x) prior to being infected had complete viral blocking in the remainder of the mosquito body 10–11 dpi. This was significantly lower (P = 0.02) than the *w*Mel.F mosquitoes which were not fed human bloodmeal (Unfed) prior to being infected. Repeat blood feeding did not have a significant effect on DENV-2 RNA copies in the remains of the Wildtype mosquito body. In replicate **B**, there was no significant change in DENV-2 RNA copies between treatment groups in both *w*Mel.F and Wildtype mosquitoes. Letters represent distinct statistical groups. Bars denote medians and each point represents an individual carcass/remainder of the mosquito body.

**Figure 7:**
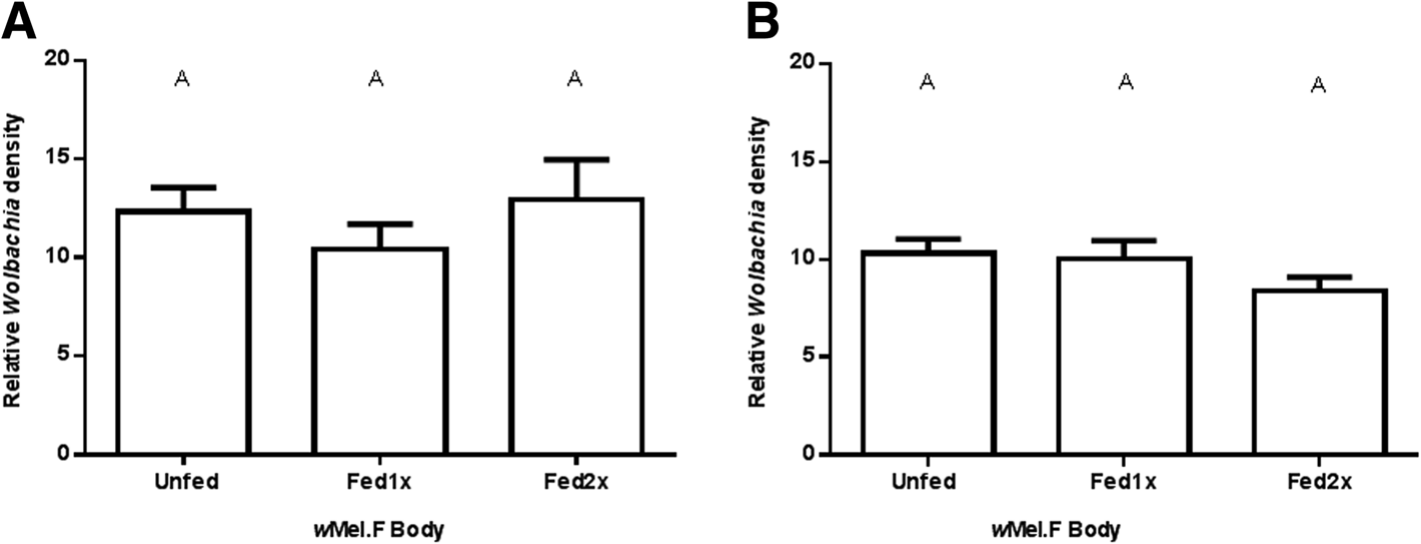
*Wolbachia* density in the remainder/carcass of the mosquito body. There was no significant change in *Wolbachia* density in *w*Mel.F remainder/carcass of the mosquito body across treatment groups in both the replicates **A** and **B**. Y-axis shows ratio of *wsp*/*Rps*17. Letters represent distinct statistical groups. Error bars are standard error of the mean of 17–22 individual remainder/carcass of the mosquito body.

Repeat feeding also had no consistent effect on DENV RNA copies in the body of *w*Mel.F mosquitoes. In replicate A (Figure 6A), there was a significant decrease in DENV RNA copies after the second bloodmeal but in replicate B (Figure 6B) viral RNA copies were fairly stable as infection rates were low. Repeat bloodmeals did not have an effect on DENV RNA copies in the body of Wildtype mosquitoes (Figure 6). *Wolbachia* density in the body also did not change significantly following repeat feeding in both replicate A and B (Figure 7). The body of the mosquito included the ovaries which are known to have a high abundance of *Wolbachia* [11,17,37] and with each oviposition some of the symbiont may have been lost to the embryos as in *Drosophila* [38] since the bacteria is concentrated in nurse cells [11] and oocytes [39]. A decrease in *Wolbachia* density in ovaries following blood feeding and oviposition was observed in *w*Flu present in *Ae. fluviatilis* [36]. There is a possibility that the presence of ovaries may have masked smaller changes in *Wolbachia* in other tissues in the body due to repeat feeding. This is not in keeping with the previous study [25] though where increases in *Wolbachia* density were seen in whole mosquitoes. Future work should focus on the carcass and ovaries separately to determine if blood feeding has an effect on *Wolbachia* density in these tissues.

Interestingly, the densities measured here in the *w*Mel.F mosquito body are far greater (~10 fold) than estimates from whole mosquitoes in the previous study [25] that reported an effect of repeat blood feeding on *Wolbachia* density. Our estimates of densities in the salivary glands and midguts were also slightly higher (1–1.5 fold). The previous and the current study differed in the use of sheep’s blood versus human blood and in the time the mosquito lines were collected from the field (roughly 2 years apart). Past work has indicated that non-human bloodmeal may be nutritionally depauperate, revealing fitness defects only when *Wolbachia* infection is present, presumably because the symbiont is competing for nutrients [40,41]. It is possible that when fed sheep’s blood *Wolbachia* are more nutritionally limited and hence have greater bursts of replication following repeat blood feeding events. If mosquitoes are reared instead on human blood that is nutritionally more appropriate for the mosquito there may be no limitation on nutrients for *Wolbachia*. While this is appealing, the explanation does not hold for our data where unfed mosquitoes have high *Wolbachia* densities to begin with and that simply do not change with subsequent feeds. Alternatively, *Wolbachia* densities may have risen in the field since release but this is difficult to ascertain without obtaining concurrent measures of density. It also indicates blood feeding may increase the density of *Wolbachia* when it is present at low densities but not when it is already at high levels.

Mosquitoes in the wild normally take small but frequent bloodmeals in one gonotrophic cycle [31-33], rarely feeding to repletion as they do under laboratory conditions where bloodmeals are readily available without disturbance or danger. In this study only two gonotrophic cycles could be studied effectively given the time required for mosquitoes to lay eggs and be interested in a subsequent meal. Hence the study design does not truly reflect feeding behaviour in the field. In future studies by intentionally interrupting feeding, mosquitoes could be made to take smaller meals, that may be digested more quickly and so a greater number of repeated feeding events could be studied. Such an approach however would come at the cost of variation in bloodmeal size between individuals.

## Conclusions

Overall, our findings indicate that at least in the *w*Mel mosquito line studied here, where *Wolbachia* densities are high in the body, that repeat feeding does not lead to subsequent increases in *Wolbachia* density nor increases in effectiveness of DENV blocking [15]. They also indicate that historical samples should be tested to determine if *Wolbachia* densities in mosquitoes have risen in the field since initial releases. Lastly, any models examining efficacy of use of *Wolbachia* as a biocontrol agent should expect *Wolbachia* density and consequently blocking ability to be constant throughout the life span of the mosquito.

## References

[1] Bhatt S, Gething WP, Brady JO, Messina PJ, Farlow WA, Moyes LC (2013). The global distribution and burden of dengue. Nature.

[2] WHO. Dengue: guidelines for diagnosis, treatment, prevention and control: New edition. Geneva: WHO press; 2009. 23762963

[3] Thomas SJ, Endy TP (2011). Critical issues in dengue vaccine development. Curr Opin Infect Dis.

[4] Zug R, Hammerstein P (2012). Still a host of hosts for *Wolbachia*: analysis of recent data suggests that 40% of terrestrial arthropod species are infected. PLoS One.

[5] Kittayapong P, Baisley KJ, Baimai V, O'Neill SL (2000). Distribution and diversity of *Wolbachia* infections in Southeast Asian mosquitoes (Diptera: Culicidae). J Med Entomol.

[6] Xi Z, Dean JL, Khoo C, Dobson SL (2005). Generation of a novel *Wolbachia* infection in *Aedes albopictus* (Asian tiger mosquito) via embryonic microinjection. Insect Biochem Mol Biol.

[7] McMeniman CJ, Lane RV, Cass BN, Fong AW, Sidhu M, Wang YF (2009). Stable introduction of a life-shortening *Wolbachia* infection into the mosquito *Aedes aegypti*. Science.

[8] Walker T, Johnson PH, Moreira LA, Iturbe-Ormaetxe I, Frentiu FD, McMeniman CJ (2011). The *w*Mel *Wolbachia* strain blocks dengue and invades caged *Aedes aegypti* populations. Nature.

[9] Serbus LR, Casper-Lindley C, Landmann F, Sullivan W (2008). The genetics and cell biology of *Wolbachia*-host interactions. Annu Rev Genet.

[10] Xi Z, Khoo CC, Dobson SL (2005). *Wolbachia* establishment and invasion in an *Aedes aegypti* laboratory population. Science.

[11] Moreira LA, Iturbe-Ormaetxe I, Jeffery JA, Lu G, Pyke AT, Hedges LM (2009). A *Wolbachia* symbiont in *Aedes aegypti* limits infection with dengue, *Chikungunya*, and *Plasmodium*. Cell.

[12] Caragata EP, Rances E, Hedges LM, Gofton AW, Johnson KN, O'Neill SL (2013). Dietary cholesterol modulates pathogen blocking by *Wolbachia*. PLoS Pathog.

[13] Rances E, Ye YH, Woolfit M, McGraw EA, O'Neill SL (2012). The relative importance of innate immune priming in *Wolbachia*-mediated dengue interference. PLoS Pathog.

[14] Pan X, Zhou G, Wu J, Bian G, Lu P, Raikhel AS (2012). *Wolbachia* induces reactive oxygen species (ROS)-dependent activation of the Toll pathway to control dengue virus in the mosquito *Aedes aegypti*. Proc Natl Acad Sci U S A.

[15] Hoffmann AA, Montgomery BL, Popovici J, Iturbe-Ormaetxe I, Johnson PH, Muzzi F (2011). Successful establishment of *Wolbachia* in *Aedes* populations to suppress dengue transmission. Nature.

[16] Iturbe-Ormaetxe I, Walker T, ON SL (2011). *Wolbachia* and the biological control of mosquito-borne disease. EMBO Rep.

[17] Dobson SL, Bourtzis K, Braig HR, Jones BF, Zhou W, Rousset F (1999). *Wolbachia* infections are distributed throughout insect somatic and germ line tissues. Insect Biochem Mol Biol.

[18] Bian G, Xu Y, Lu P, Xie Y, Xi Z (2010). The endosymbiotic bacterium *Wolbachia* induces resistance to dengue virus in *Aedes aegypti*. PLoS Pathog.

[19] Frentiu FD, Robinson J, Young PR, McGraw EA, O'Neill SL (2010). *Wolbachia*-mediated resistance to dengue virus infection and death at the cellular level. PLoS One.

[20] Lu P, Bian G, Pan X, Xi Z (2012). *Wolbachia* induces density-dependent inhibition to dengue virus in mosquito cells. PLoS Negl Trop Dis.

[21] Ikeda T, Ishikawa H, Sasaki T (2003). Regulation of *Wolbachia* density in the Mediterranean flour moth, *Ephestia kuehniella*, and the almond moth, *Cadra cautella*. Zoolog Sci.

[22] Kondo N, Shimada M, Fukatsu T (2005). Infection density of *Wolbachia* endosymbiont affected by co-infection and host genotype. Biol Lett.

[23] Mouton L, Henri H, Charif D, Bouletreau M, Vavre F (2007). Interaction between host genotype and environmental conditions affects bacterial density in *Wolbachia* symbiosis. Biol Lett.

[24] Dutton TJ, Sinkins SP (2004). Strain-specific quantification of *Wolbachia* density in *Aedes albopictus* and effects of larval rearing conditions. Insect Mol Biol.

[25] Frentiu FD, Zakir T, Walker T, Popovici J, Pyke AT, van den Hurk A (2014). Limited dengue virus replication in field-collected *Aedes aegypti* mosquitoes infected with *Wolbachia*. PLoS Negl Trop Dis.

[26] Black WC, Bennett KE, Gorrochotegui-Escalante N, Barillas-Mury CV, Fernandez-Salas I, de Lourdes MM (2002). Flavivirus susceptibility in *Aedes aegypti*. Arch Med Res.

[27] Warrilow D, Northill JA, Pyke A, Smith GA (2002). Single rapid TaqMan fluorogenic probe based PCR assay that detects all four dengue serotypes. J Med Virol.

[28] Thellin O, Zorzi W, Lakaye B, De Borman B, Coumans B, Hennen G (1999). Housekeeping genes as internal standards: use and limits. J Biotechnol.

[29] Cook PE, Hugo LE, Iturbe-Ormaetxe I, Williams CR, Chenoweth SF, Ritchie SA (2006). The use of transcriptional profiles to predict adult mosquito age under field conditions. Proc Natl Acad Sci U S A.

[30] Ye YH, Woolfit M, Rances E, O'Neill SL, McGraw EA (2013). *Wolbachia*-associated bacterial protection in the mosquito *Aedes aegypti*. PLoS Negl Trop Dis.

[31] Scott TW, Amerasinghe PH, Morrison AC, Lorenz LH, Clark GG, Strickman D (2000). Longitudinal studies of *Aedes aegypti* (Diptera: Culicidae) in Thailand and Puerto Rico: blood feeding frequency. J Med Entomol.

[32] Scott TW, Chow E, Strickman D, Kittayapong P, Wirtz RA, Lorenz LH (1993). Blood-feeding patterns of *Aedes aegypti* (Diptera: Culicidae) collected in a rural Thai village. J Med Entomol.

[33] Scott TW, Clark GG, Lorenz LH, Amerasinghe PH, Reiter P, Edman JD (1993). Detection of multiple blood feeding in *Aedes aegypt*i (Diptera: Culicidae) during a single gonotrophic cycle using a histologic technique. J Med Entomol.

[34] Koella JC, Sorense FL (2002). Effect of adult nutrition on the melanization immune response of the malaria vector *Anopheles stephensi*. Med Vet Entomol.

[35] Chun J, Riehle M, Paskewitz SM (1995). Effect of mosquito age and reproductive status on melanization of sephadex beads in *Plasmodium*-refractory and -susceptible strains of *Anopheles gambiae*. J Invertebr Pathol.

[36] Baton LA, Pacidonio EC, Goncalves DS, Moreira LA (2013). *w*Flu: characterization and evaluation of a native *Wolbachia* from the mosquito *Aedes fluviatilis* as a potential vector control agent. PLoS One.

[37] Zouache K, Voronin D, Tran-Van V, Mousson L, Failloux AB, Mavingui P (2009). Persistent *Wolbachia* and cultivable bacteria infection in the reproductive and somatic tissues of the mosquito vector *Aedes albopictus*. PLoS One.

[38] McCall K (2004). Eggs over easy: cell death in the *Drosophila* ovary. Dev Biol.

[39] Ferree PM, Frydman HM, Li JM, Cao J, Wieschaus E, Sullivan W (2005). *Wolbachia* utilizes host microtubules and Dynein for anterior localization in the *Drosophila* oocyte. PLoS Pathog.

[40] Caragata EP, Rances E, O'Neill SL, McGraw EA (2014). Competition for amino acids between *Wolbachia* and the mosquito host, *Aedes aegypti*. Microb Ecol.

[41] McMeniman CJ, Hughes GL, O'Neill SL (2011). A *Wolbachia* symbiont in *Aedes aegypti* disrupts mosquito egg development to a greater extent when mosquitoes feed on nonhuman versus human blood. J Med Entomol.

